# On Certain Differences in the Composition of the Blood in the Male and Female

**Published:** 1845-08

**Authors:** 


*On certain Differences in the Composition of the Blood in the Male and Female.*—MM. Becquerel and Rodier read a very elaborate memoir "on the Composition of the Blood in Health and in Disease," before the Royal Academy of Sciences, in the course of last November. As it must always be of the first importance to determine the normal condition of any of the fluids of the body, before we attempt to ascertain its morbid alterations, their remarks on the relative constitution of the blood in healthy adults of the two sexes may be deemed acceptable. The proportions given in the following table were determined by taking the average or medium figures obtained in a variety of experiments.

| | *Man.* | *Woman.* |
|---|---|---|
| Water - - - - - - | 779 | 791,1 |
| Globules - - - - - | 141,1 | 17,2 |
| Albumen - - - - - - | 69,4 | 70,5 |
| Fibrine - - - - - | 2,2 | 2,2 |
| Extractive matters and free salts - | 6,8 | 7,4 |
| Fatty matters - - - - - | 1,6 | 1,620 |
| Seroline - - - - - - | 0,020 | 0,020 |
| Phosphorated fatty matter - | 0,488 | 0,464 |
| Cholesterine - - - - - | 0,088 | 0,090 |
| Soap - - - - - | 0,004 | 0,046 |
| In 100 parts of calcined blood. | | |
| Cloride of sodium - - - - | 3,1 | 3,9 |
| Soluble salts - - - - - | 2,5 | 2,9 |
| Phosphates . - . - | 0,334 | 0,354 |
| Iron - - - - - - | 0,565 | 0,541 |
| Density of the defibrinated blood | 1060,2 | 1057,5 |
| of the serum | 1028 | 1027,4 |

By comparing the two columns in this table, we find that certain very noticeable differences exist between the blood of the male and that of the female, in a state of health. The density of the defibrinated fluid is greater in the former, and consequently contains a larger quantity of soluble matters ; the proportion of water too is decidedly less. The quantity of the colored globules is considerably greater in the blood of the male than in that of the female : this is perhaps the most important, and indeed it is the fundamental, difference in the blood of the two sexes. In the female, the *minimum* number was 113, the *maximum* was 137, and the *medium* 127 ; (?) whereas in the case of the male, the *minimum* was 131, the *maximum* 151, and the *medium*

141.* The proportion of the Fibrine, and also that of the Albumen, was found to be very nearly the same in both sexes. The proportion of the iron present in the blood is always commensurate with that of the red globules.

MM. Becquerel and Rodier are of opinion that the function of *menstruation* exercises a marked influence on the proportion of the red globules in the blood of the female. In the girl, before this function has properly commenced, the relative quantity is below the normal standard; when the secretion is fairly established, it (the quantity) rises up to 127 or even higher; and this state of things continues until about the critical period of life, when menstruation ceases: then the proportion of the red globules falls considerably below this mark.

*Pregnancy* also has a very decided influence on the condition of the blood; the red globules and the Albumen become diminished, and the fibrine, phosphorated fatty matter, and the water slightly increased.—*Med. Chir. Rev. from Ency. des Sci. Med.*

* This proportion is considerably higher than that (viz. 127) assumed by Andral and other hematological enquirers, as the standard of health; while the proportion of the fibrine in our table is lower than that (3) in theirs.

